# Dissociation and emotional dysregulation in pathological personalities related to the fear of SARS-COV-2: a case report

**DOI:** 10.1192/j.eurpsy.2022.1348

**Published:** 2022-09-01

**Authors:** R. Sousa, J. Brás, A. Costa, R. Vaz, J. Martins, D. Teixeira, A. Marques, J. Abreu, E. Almeida, N. Cunha

**Affiliations:** Centro Hospitalar Tondela-Viseu , Departamento De Psiquiatria E Saúde Mental, Viseu, Portugal

**Keywords:** personality, fear, PTSD, covid

## Abstract

**Introduction:**

The COVID-19 pandemic represented a serious strain on the mental health resilience worldwide. Implementation of restrictive rules implied the disruption of social networks, eliciting emotional exhaustion and intense response to fear. This was amplified by media spread of panic and fake news, representing risk factors for post traumatic stress disorder (PTSD). Fear can be dangerous, especially accounting premorbid psychopathological vulnerability, such as pathological personality traits. Emotional dysregulation increases fear levels, mediated by the relationship between emotional dysregulation and lack of tolerance.

**Objectives:**

Clinical case presentation of patient who developed dissociative and behavioral symptoms following COVID-19 infection. Bibliographic research.

**Methods:**

Bibliographic research using Pubmed®. Clinical file consultation and patient interviews.

**Results:**

Heightened psychophysiological reactivity can result from the persistent fear experienced during a traumatic event and repeated memories related to it, leading to a sensitization of the response to fear. We present 57 year-old female patient, admitted to the COVID ward after trying to escape from home isolation due to positivity to COVID-19. In the hospital setting she developed dissociative symptoms, trying to escape from the ward and infect other people.

**Conclusions:**

Intense fear responses to COVID-19 are likely explained by poor emotion regulation capacities as well as dissociative mechanisms. Studies have shown that this pandemic was experienced as a real traumatic event and some studies have found that it may lead to the development of PTSD. Pathological personality is positively related to PTSD symptoms, attributable to higher levels of mood instability, cognitive/perceptual disorders, interpersonal dysfunctions and negative affection.

**Disclosure:**

No significant relationships.

